# Analysis of the Clinical Efficacy and Molecular Mechanism of Xuefu Zhuyu Decoction in the Treatment of COPD Based on Meta-Analysis and Network Pharmacology

**DOI:** 10.1155/2022/2615580

**Published:** 2022-11-26

**Authors:** Yu Hu, Yunxi Lan, Qiqi Ran, Qianrong Gan, Wei Huang

**Affiliations:** School of Basic Medical Sciences, Chengdu University of Traditional Chinese Medicine, Chengdu 611137, China

## Abstract

**Background:**

Chronic obstructive pulmonary disease (COPD) is becoming a major public health burden worldwide. It is urgent to explore more effective and safer treatment strategy for COPD. Notably, Xuefu Zhuyu Decoction (XFZYD) is widely used to treat respiratory system diseases, including COPD, in China.

**Objective:**

This study is aimed at comprehensively evaluating the therapeutic effects and molecular mechanism of XFZYD on COPD.

**Methods:**

Original clinical studies were searched from eight literature databases. Meta-analysis was conducted using the Review Manager software (version 5.4.1). Network pharmacology and molecular docking experiments were utilized to explore the mechanisms of action of XFZYD.

**Results:**

XFZYD significantly enhanced the efficacy of clinical treatment and improved the pulmonary function and hypoventilation of COPD patients. In addition, XFZYD significantly improved the hypercoagulability of COPD patients. The subgroup analysis suggested that XFZYD exhibited therapeutic effects on both stable and acute exacerbation of COPD. XFZYD exerted its therapeutic effects on COPD through multicomponent, multitarget, and multipathway characteristics. The intervention of the PI3K-AKT pathway may be the critical mechanism.

**Conclusion:**

The application of XFZYD based on symptomatic relief and supportive treatment is a promising clinical decision. More preclinical and clinical studies are still needed to evaluate the safety and therapeutic effects of long-term use of XFZYD on COPD.

## 1. Introduction

Chronic obstructive pulmonary disease (COPD) is a chronic inflammatory disease of the respiratory system induced by long-term exposure to risk factors such as tobacco smoke, toxic gases, or polluted air [1]. COPD is mainly characterized by progressive involvement of the airways, alveoli, and microvasculature, leading to irreversible airflow limitation [2]. Emphysema and chronic bronchitis are two typical phenotypes of COPD, but are usually present in one patient simultaneously [3, 4]. Currently, COPD has a global prevalence of approximately 328 million and has become the third leading death cause after ischemic heart disease and tumors [5, 6]. Smoking is considered as a vital risk factor of COPD. Although the proportion of population that smokes has declined globally, environmental particulate matter pollution and occupational second-hand smoke exposure have increased dramatically over the past 25 years, which may become major risk factors for COPD in the future [7, 8]. As a result, the prevalence of COPD is still increasing and is forecasted to become the fifth largest global disease burden by 2030 [9]. Additionally, age is an important risk factor for COPD [10]. The incidence risk of COPD tends to increase exponentially with age, with the middle-aged and elderly population being the main groups affected [11]. Obviously, the prevalence and mortality of COPD are gradually increasing and the burden of disease continues to grow with the aging of global society [12, 13]. There is no doubt that COPD is increasingly becoming a serious global public health concern [14].

Although the treatment of COPD has evolved and improved significantly over the past 20 years, there is no known cure for COPD but only options to improve symptoms and support health of patients [3, 15]. Moreover, it is still dependent on bronchodilators and corticosteroids, which have significant limitations. The long-term use of corticosteroids increases the risk of adverse events in patients [16]. At the same time, the progression of emphysema cannot be inhibited by such drugs [17]. In addition, COPD patients who suffer from viral or bacterial infections or increased bacterial load in the airways, resulting in acute worsening of respiratory symptoms, are referred to as acute exacerbation of COPD (AECOPD) [9, 18]. It is noticed that efficacy of corticosteroids in AECOPD is very limited although they are the main anti-infective drugs used in COPD [19]. Therefore, there is an urgent need for alternative, more effective, and safer treatments for COPD to alleviate clinical symptoms, stop disease progression, and reverse the disease process [20].

**Figure 1:**
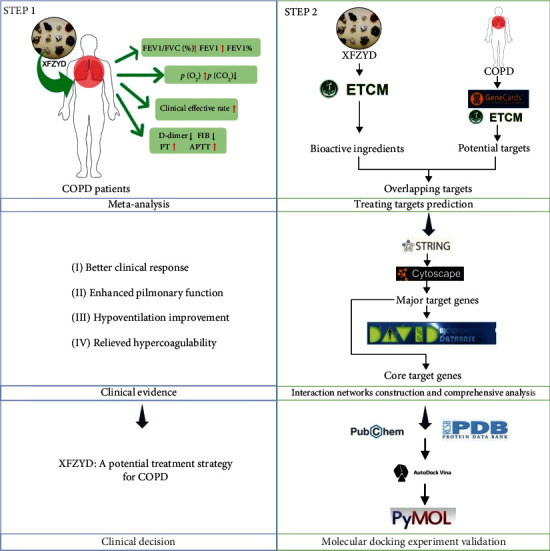
The theme of the present study.

As an important component of complementary alternative medicine, traditional Chinese medicine is believed to exert its pharmacological effects through multicomponent, multitarget, and multipathway characteristics, thus offering significant advantages in treating many chronic diseases with fewer adverse events [21]. Xuefu Zhuyu Decoction (XFZYD) is a formula from *Corrections on the Errors of Medical Works* (*Yi Lin Gai Cuo*) of Wang Qingren in the Qing Dynasty, comprising the dried ripe seed of *Prunus persica* (L.) Batsch (*Tao Ren*), the dried flower of *Carthamus tinctorius* L. (*Hong Hua*), the dried root of *Angelica sinensis* (Oliv.) Diels (*Dang Gui*), the fresh or dried tuberous root of *Rehmannia glutinosa* (Gaertn.) DC. (*Sheng Di*), the dried rhizome of *Conioselinum anthriscoides “Chuanxiong”* (*Chuan Xiong*), the dried root of *Paeonia lactiflora* Pall. (*Chi Shao*), the dried root of *Bupleurum chinense* DC. (*Chai Hu*), the dried unripe fruit of *Citrus × aurantium* L. (*Zhi Qiao*), the dried root of *Platycodon grandiflorus* (Jacq.) A.DC. (*Jie Geng*), the dried root of *Achyranthes bidentata* Blume (*Niu Xi*), and the dried root and rhizome of *Glycyrrhiza glabra* L. (*Gan Cao*), which has been used to treat cardiovascular and microvascular diseases in clinical practice [22, 23]. Several systematic evaluations have assessed the efficacy in XFZYD in chronic diseases such as uterine fibroids, pulmonary fibrosis, and coronary heart disease with positive results [24–26]. Meanwhile, a large number of clinical observational studies have focused on its therapeutic effects on COPD [27]. However, to date, there is still a lack of systematic evaluation of the therapeutic effects of XFZYD on COPD. Moreover, the specific molecular mechanisms of XFZYD in the treatment of COPD are still unclear. In the present study, the efficacy and safety of XFZYD in COPD were investigated by meta-analysis, and the therapeutic advantages of XFZYD in the stable COPD and AECOPD were further measured by subgroup analysis. Then, the network pharmacology and molecular docking experiments were used for exploring the mechanism of action of XFZYD in the treatment of COPD (Figure 1).

## 2. Materials and Methods

### 2.1. Assessment of Efficacy and Safety of XFZYD Based on Meta-Analysis

This study followed the Cochrane criteria and the Preferred Reporting Items for Systematic Review and Meta-Analysis (PRISMA) statement guidelines [28]. Moreover, this meta-analysis protocol was registered with the PROSPERO (CRD42022304855).

#### 2.1.1. Study Selection

The databases, including Cochrane library, PubMed, Embase, Web of Science (WOS), China Academic Journals (CNKI), Wan-fang database, Chinese biomedical literature service system (CBM), and Chinese Science and Technology Journals (CQVIP), were searched systematically before January 2022. No language and region restrictions were considered for original studies. The search terms were “Chronic obstructive pulmonary disease OR COPD” and “Xuefu Zhuyu OR Xuefuzhuyu OR XueFuZhuYu.” The detailed searching strategies are accessible in Supplementary Table 1.

#### 2.1.2. Inclusion and Exclusion Criteria

The PICOS criteria were used for the inclusion and exclusion of original studies. The inclusion criteria are as follows: (1) participants: patients diagnosed with COPD using definite diagnostic criteria, including stable COPD and AECOPD; (2) intervention: XFZYD combined with symptomatic supportive treatments (only the whole formula of XFZYD used could be included); (3) control: symptomatic relief and supportive treatment alone; (4) outcomes: the pulmonary function indicators and clinical effective rate are the primary outcomes, the blood gas analysis results, coagulation indexes, and adverse events are the secondary outcomes; (5) study: randomized controlled trials (RCTs).

The exclusion criteria are as follows: (1) original study without any diagnostic criteria; (2) patients with COPD combined with other critical illnesses; (3) other TCM treatments, such as acupuncture and Tai Chi, were used in the treatment and control groups; (4) case reports, retrospective studies, reviews, systematic reviews, and other non-RCT studies.

#### 2.1.3. Data Extraction

The original studies were reviewed independently by two authors (Y.L. and Q.R.). Then, the specific data were extracted, including first author, publication year, numbers of patients, average age, disease stage, outcomes, and adverse events. Differences were resolved by the discussion between two authors; differences which could not be resolved were settled by the third author (Q.G.).

#### 2.1.4. Risk of Bias Assessment

The revised Cochrane risk-of-bias tool for randomized trials (RoB 2) was used to assess the risk of bias [29]. Specifically, five domains including randomization process, deviations from the intended interventions, missing outcome data, measurement of the outcome, and selection of the reported result were judged as low risk of bias, some concerns, or high risk of bias based on the summaries of answers to signaling questions. Finally, an overall bias, which corresponds to the worst risk of bias in any of the domains, for every included study was judged.

#### 2.1.5. Outcomes

Impaired pulmonary function is a critical diagnostic standard of COPD as well as an important indication of the prognosis of patients [30]. As a result, the pulmonary function indexes, including the forced expiratory volume in one second/forced vital capacity (FEV1/FVC (%)), the volume of forced expiratory volume in one second (FEV1), and the percentage of forced expiratory volume in one second (FEV1%), were settled as the primary outcomes. Similarly, the clinical effective rate was also identified as primary outcomes since it tends to be comprehensive for the evaluation of the therapeutic effects of treatment strategies. Then, the blood gas analysis results, including arterial partial pressure of oxygen (*p*(O2)) and the arterial partial pressure of carbon dioxide (*p*(CO2)), coagulation indexes, comprising D-dimer, fibrinogen (FIB), prothrombin time (PT), and activated partial thromboplastin time (APTT), and adverse events were the secondary outcomes.

In order to clarify the therapeutic advantage of XFZYD of different stages of COPD, we set up the subgroup analysis of these outcomes of stable COPD and AECOPD.

#### 2.1.6. Statistical Analysis

The Review Manager (RevMan) software (version 5.4.1) was used to perform the meta-analysis. The weighted mean difference (WMD) or standardized mean difference (SMD) with a 95% confidence interval (CI) was used to assess the continuous variables. The risk ratio (RR) or risk difference (RD) with a 95% CI were applied to analyze the dichotomous variables. Heterogeneity was estimated by Cochran's *Q* test and assessed using *I*^2^. When *I*^2^ was less than 50%, it was considered homogeneous and is evaluated by a fixed-effects model. When *I*^2^ was greater than 50%, it is considered heterogeneous and is assessed using a random-effects model, and the sensitivity analysis would be conducted to assess the sources for heterogeneity in primary outcomes. Finally, the funnel plots were created to check for publication bias. The Grading of Recommendations Assessment, Development, and Evaluation (GRADE) profiler software (version 3.6) was applied to evaluate the quality of the clinical evidence, which mainly have four grades, high, moderate, low, and very low [31].

### 2.2. Exploration of the Molecular Mechanisms of XFZYD for COPD Based on Network Pharmacology

#### 2.2.1. Collection of the Bioactive Ingredients and Potential Targets of XFZYD

The bioactive ingredients of XFZYD were searched from the Encyclopedia of Traditional Chinese Medicine (ETCM, http://www.tcmip.cn/ETCM/) database [32]. Ingredients in ETCM database were mainly assessed by the quantitative estimate of drug-likeness (QED) score based on models in the Pipeline Pilot ADMET collection. Therefore, the ingredients with moderate to good-likeness (QED > 0.49) were considered as bioactive ingredients. Then, the targets of bioactive components, which were predicted by the MedChem Studio (version 3.0; Simulations Plus, Lancaster, CA, 2012) according to the structural and functional similarities of drugs, were also collected from ETCM database [33]. The targets with high similarity (structural similarity score > 0.80) were obtained for further analysis.

#### 2.2.2. Prediction of the Targets of COPD

Firstly, the targets of COPD were searched from the ETCM database, including the results of Therapeutic Target Database (TTD, http://db.idrblab.net/ttd/) and Human Phenotype Ontology (HPO, https://hpo.jax.org/app/) databases. Then, the targets were searched from GeneCards (https://www.genecards.org/) database. The targets in GeneCards that exceed the median of all target relevance score were retained as potential targets for COPD. Finally, the targets from ETCM and GeneCards were combined to further investigation.

#### 2.2.3. Construction of the Protein-Protein Interaction (PPI) Network and Target Analysis

The overlapping targets of XFZYD and COPD were input to STRING (https://cn.string-db.org/) database to perform the PPI network with organism limited to Homo sapiens. The minimum required interaction score was set at the highest confidence (>0.900). Moreover, the independent unconnected nodes are hidden. Then, the network from STRING was exported to Cytoscape (version 3.9.1) for visualization and analysis [34]. The node targets whose three topological parameters (betweenness centrality, closeness centrality, and degree) exceeding median were considered as the major targets of XFZYD on COPD. Finally, major node targets with all three topology parameters exceeding median were identified as core node targets.

#### 2.2.4. Gene Ontology (GO) and Kyoto Encyclopedia of Genes and Genomes (KEGG) Pathway Enrichment Analysis

The GO and KEGG enrichment analysis were carried out to explore the molecular mechanisms of XFZYD on COPD by the Database for Annotation, Visualization, and Integrated Discovery (DAVID, https://david.ncifcrf.gov/) [35, 36]. The top 10 GO and KEGG enrichment results with *p* values less than 0.05 were retained and input to bioinformatics (https://www.bioinformatics.com.cn), a free online platform for data analysis and visualization, for visualization.

#### 2.2.5. Construction of Related Networks

Two networks were constructed and visualized by Cytoscape: (1) the PPI network and (2) herb-bioactive ingredient-major target-pathway network.

#### 2.2.6. Molecular Docking

According to the analysis results of the herb-bioactive ingredient-major target-pathway network, the top 3 ingredients of XFZYD were selected for molecular docking. The structures of ingredients were downloaded from PubChem (https://pubchem.ncbi.nlm.nih.gov/). Then, the target genes for molecular docking need to meet two requirements: (1) targets must be core node targets in PPI network and (2) the top 3 targets in herb-bioactive ingredient-major target-pathway network sorted by degree value. The structures of target proteins were obtained from Protein Data Bank (PDB, https://www.rcsb.org/) database. The molecular docking was conducted by AutoDock Vina (version 1.2.0) and visualized by PyMOL (version 2.5) [37].

## 3. Results

### 3.1. The Efficacy and Safety of XFZYD on COPD

#### 3.1.1. Study Research and Study Characteristics

**Figure 2:**
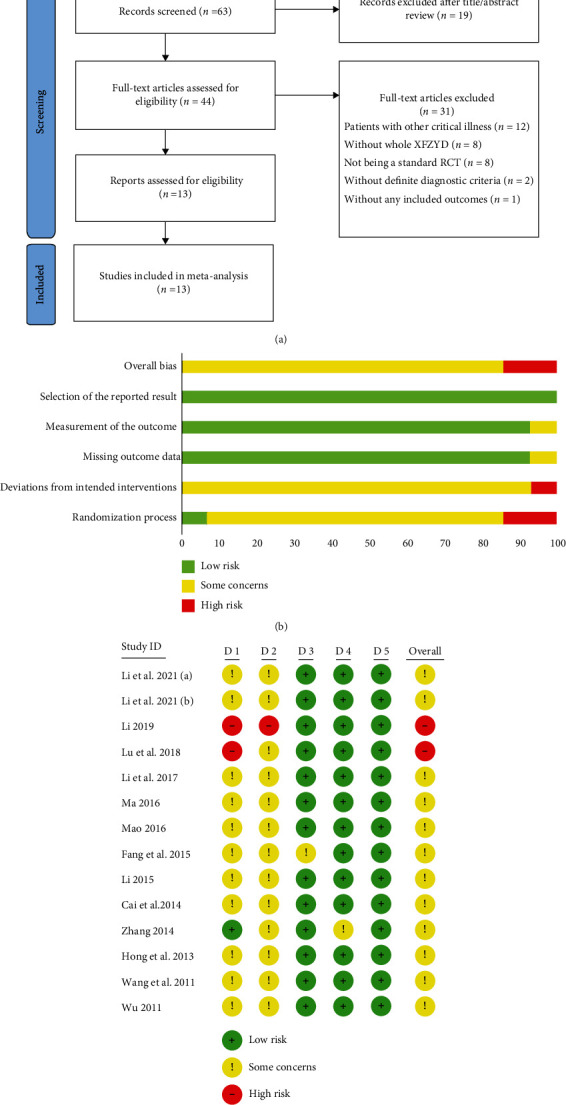
(a) The study selection process, (b) results of the summary of risk of bias, and (c) the risk of bias of each original study.

A total of 111 relevant studies were searched from 8 databases. After removal of duplicate articles, 63 studies were remained. Then, 44 articles were retained after the title/abstract reviewing. Finally, 31 articles were excluded after the full-text reviewing, and the 13 remaining articles were included for this meta-analysis (Figure 2(a)) [27, 38–49].

The general characteristics of the included studies are shown in Table 1. The specific dosages of XFZYD and the treatment strategies of the control group in each study are shown in Supplementary Table 2. The published articles ranged from 2011 to 2021. 893 patients were included, with 448 in the treatment group and 445 in the control group. All of the studies were conducted in China. Notably, 1 included article was divided into two separate studies (Li et al. 2021a and Li et al. 2021b) for meta-analysis because both stable and acute exacerbation studies and different control groups were established [27].

#### 3.1.2. Risk of Bias Evaluation

The RoB 2 was applied to assess the risk of bias of included studies (Figures 2(b) and 2(c)). For overall bias, a total of 12 included studies were assessed as some concerns, and another 2 trials were judged as high risk. The poor results of the overall risk of bias assessment were mainly due to the absence of random assignment and allocation concealment details in the original studies, especially in the clinical trials conducted by Li et al. [42] and Liu et al. [45], which suggested the low quality of original studies.

#### 3.1.3. Meta-Analysis of Pulmonary Function Indexes

**Figure 3:**
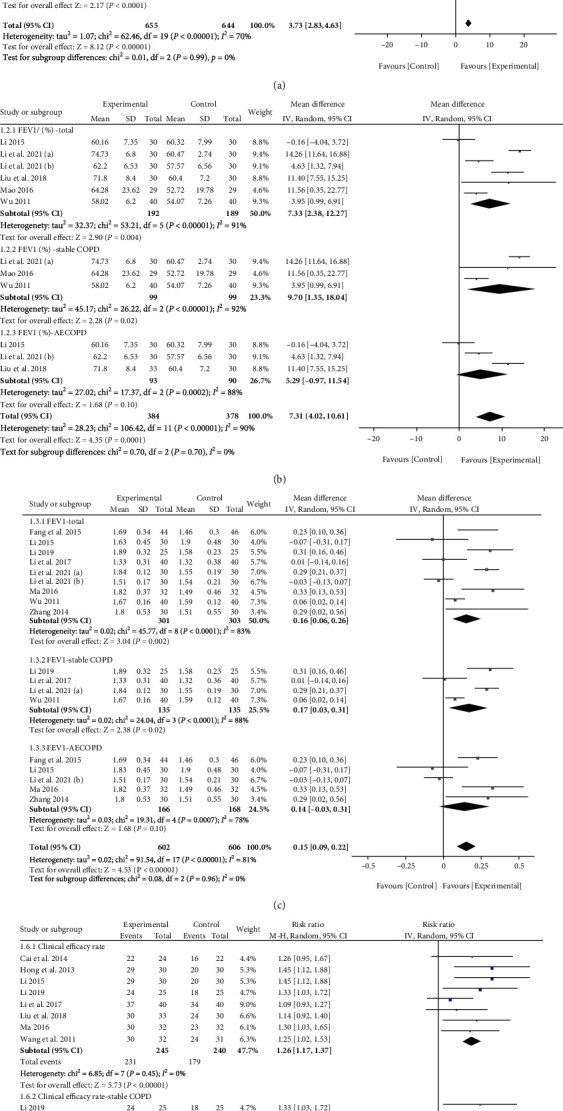
The meta-analysis results of primary outcomes. (a) The forest plot of FEV1/FVC (%). (b) The forest plot of FEV1%. (c) The forest plot of FEV1. (d) The forest plot of clinical effective rate.

Ten studies reported on the FEV1/FVC (%) of XFZYD treatment. The results showed that the FEV1/FVC (%) in the experimental group was significantly higher than that in the control group (WMD = 4.12; 95% CI: 2.09, 6.15; *p* < 0.0001; *I*^2^ = 71%) (Figure 3(a)). Due to the appreciable heterogeneity, the sensitivity analysis was conducted on ten studies by excluding them one by one. The result showed that the trials conducted by Li et al. [47], Li et al. [27], and Mao [38] are the source of heterogeneity; *I*^2^ decreased from 71% to 29% after their exclusion, with *p* < 0.00001 (Supplementary Figure 1). Then, the subgroup analysis was performed based on the different disease stages of COPD. The results showed that the XFZYD significantly improved FEV1/FVC (%) in stable COPD (WMD = 4.37; 95% CI: 0.16, 8.57; *p* = 0.04; *I*^2^ = 83%) (Figure 3(a)) and AECOPD (WMD = 4.19; 95% CI: 1.16, 7.23; *p* = 0.007; *I*^2^ = 62%) (Figure 3(a)).

Six studies evaluated the FEV1% after the XFZYD treatment. The meta-analysis results suggested that compared with that in the control group, the FEV1% was significantly increased in the experimental group (WMD = 7.33, 95% CI: 2.38, 12.27; *p* = 0.004; *I*^2^ = 91%) (Figure 3(b)). The heterogeneity remained high under the investigation of sensitivity analysis by excluding the trials one by one, resulting in the inability to identify the source of heterogeneity. Regarding the subgroup analysis, the FEV1% was significantly enhanced in the experimental group in the stable COPD (WMD = 9.70, 95% CI: 1.35, 18.04; *p* = 0.02; *I*^2^ = 92%) (Figure 3(b)). However, there was no significant difference in the AECOPD after the XFZYD treatment (WMD = 5.29, 95% CI: -0.97, 11.54; *p* = 0.10; *I*^2^ = 88%) (Figure 3(b)).

FEV1 was reported in nine included studies. The results showed that XFZYD could significantly improve the FEV1 level (WMD = 0.16, 95% CI: 0.06, 0.26; *p* = 0.002; *I*^2^ = 83%) (Figure 3(c)). In light of the obvious heterogeneity, the sensitivity analysis was conducted. However, the results implied that the heterogeneity was still large. By setting the subgroup analysis, we found that the FEV1 level in the experimental group was higher than that in the control group at stable COPD (WMD = 0.17, 95% CI: 0.03, 0.31; *p* = 0.02; *I*^2^ = 88%) (Figure 3(c)). Nevertheless, there was no difference in FEV1 level between the experimental group and the control group on AECOPD (WMD = 0.14, 95% CI: -0.03, 0.31; *p* = 0.10; *I*^2^ = 79%) (Figure 3(c)).

#### 3.1.4. Meta-Analysis of Clinical Effective Rate

As another primary outcome, the clinical effective rate was reported in eight included studies. Assessed by a fixed-effects model, the meta-analysis results showed that the clinical effective rate of the experimental group was significantly higher than that of the control group (RR = 1.26, 95% CI: 1.17, 1.37; *p* < 0.00001; *I*^2^ = 0%) (Figure 3(d)). Moreover, the subgroup analysis implied that XFZYD treatment could significantly enhance the clinical effective rate in both stable COPD (RR = 1.17, 95% CI: 1.02, 1.35; *p* = 0.02; *I*^2^ = 45%) (Figure 3(d)) and AECOPD (RR = 1.26, 95% CI: 1.15, 1.37; *p* = 0.00001; *I*^2^ = 23%) (Figure 3(d)).

#### 3.1.5. Meta-Analysis of Blood Gas Indexes

**Figure 4:**
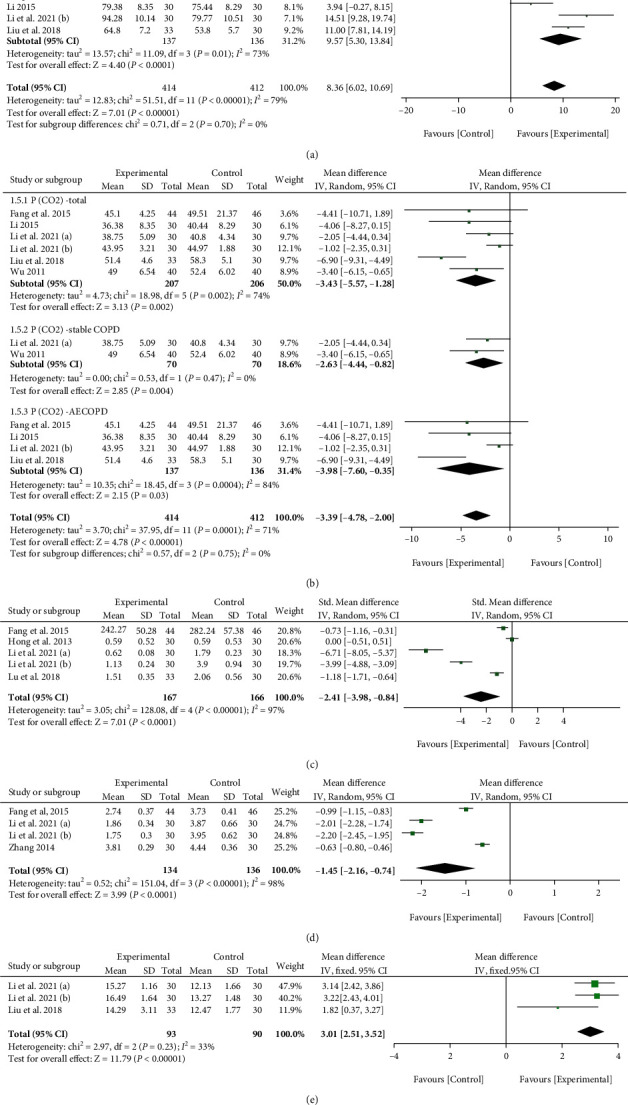
The meta-analysis results of secondary outcomes. (a) The forest plot of *p*(O_2_). (b) The forest plot of *p*(CO_2_). (c) The forest plot of D-dimer. (d) The forest plot of FIB. (e) The forest plot of PT. (f) The forest plot of APTT.

A total of six studies reported on the level of *p*(O2). Meta-analysis suggested that the level of *p*(O_2_) was higher in the experimental group compared to the control group (WMD = 8.38, 95% CI: 4.88, 11.88; *p* < 0.00001; *I*^2^ = 81%) (Figure 4(a)). Then, the subgroup analysis also showed that the *p*(O_2_) level could be restored after XFZYD treatment both in stable COPD (WMD = 6.37, 95% CI: 0.21, 12.52; *p* = 0.04; *I*^2^ = 88%) (Figure 4(a)) and AECOPD (WMD = 9.57, 95% CI: 5.30, 13.84; *p* < 0001; *I*^2^ = 73%) (Figure 4(a)).

Six studies reviewed *p*(CO2) level. Our meta-analysis results suggested that XFZYD could exhibit a decreasing effect on *p*(CO_2_) level (WMD = −3.43, 95% CI: -5.57, -1.28; *p* = 0.002; *I*^2^ = 74%) (Figure 4(b)). Similarly, XFZYD was effective in reducing the level of *p*(CO_2_) in both stable COPD (WMD = −2.63, 95% CI: -4.44, -0.82; *p* = 0.004; *I*^2^ = 0%) (Figure 4(b)) and AECOPD (WMD = −3.98, 95% CI: -7.60, -0.35; *p* = 0.03; *I*^2^ = 84%) (Figure 4(b)).

#### 3.1.6. Meta-Analysis of Coagulation Indexes

Four coagulation indexes including FIB, D-dimer, PT, and APTT were evaluated in this meta-analysis. The results showed that after XFZYD treatment, the D-dimer (SMD = −2.41, 95% CI: -3.98, -0.84; *p* = 0.003; *I*^2^ = 97%) (Figure 4(c)), FIB (WMD = −1.45, 95% CI: -2.16, -0.74; *p* < 0.0001; *I*^2^ = 98%) (Figure 4(d)), and PT (WMD = 3.01, 95% CI: 2.51, 3.52; *p* < 0.00001; *I*^2^ = 33%) (Figure 4(e)) levels were significantly improved. However, there was no difference on APTT level after the XFZYD treatment (WMD = 1.46, 95% CI: -1.15, 4.07; *p* = 0.27; *I*^2^ = 89%) (Figure 4(f)).

#### 3.1.7. Meta-Analysis of Adverse Events

**Figure 5:**
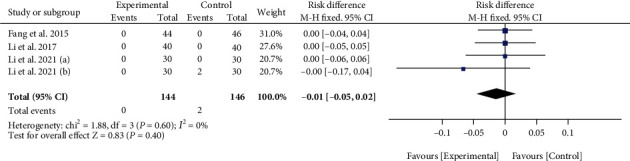
The meta-analysis results of adverse events.

Among the included 14 studies, only 4 studies reported the adverse events. Notably, three of these studies reported no adverse events in either the experimental group or the control group, and the results showed a nonsignificant difference between the experimental group and the control group (RD = −0.01, 95% CI: -0.05, 0.02; *p* = 0.40; *I*^2^ = 0%) (Figure 5).

#### 3.1.8. Publication Bias

**Figure 6:**
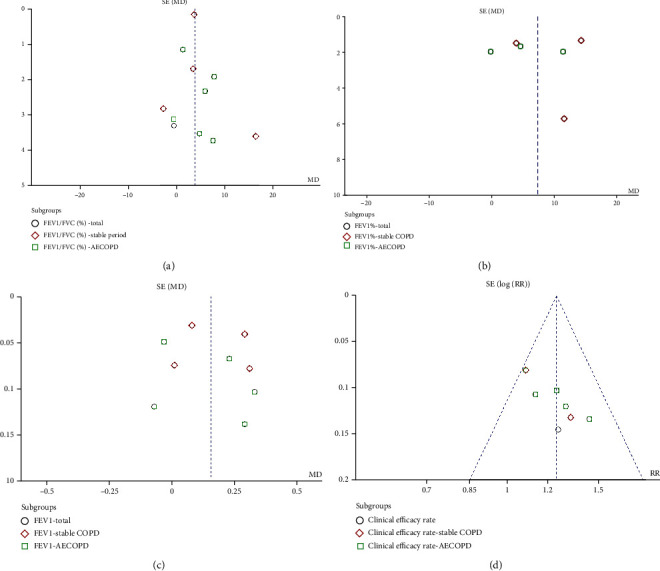
The publication bias assessment of primary outcomes. (a) The publication bias of FEV1/FVC (%). (b) The publication bias of FEV1%. (c) The publication bias of FEV1. (d) The publication bias of clinical effective rate.

The publication bias of primary outcomes was assessed by funnel plots. The funnel plots were asymmetrical, which showed a possible publication bias (Figure 6).

#### 3.1.9. Quality of Evidence

The quality of the evidence was generally low, with only two indicators of clinical effective rate and PT judged to be of moderate quality (Supplementary Figure 2). The reason for the downgrading of the evidence quality is primarily from the worrisome methodology of included studies, which is consistent with the factors influencing the risk of bias assessment.

### 3.2. The Molecular Mechanisms of XFZYD on COPD Based on Network Pharmacology

#### 3.2.1. The Bioactive Ingredients and Potential Targets of XFZYD and Treating Targets of COPD

A total of 499 ingredients were obtained from the ETCM database. After the assessment of QED score, 248 components were retained and identified as bioactive ingredients of XFZYD. Then, 115 ingredients which had high similarity targets were chosen. Finally, 115 bioactive ingredients with 578 potential targets were left.

**Figure 7:**
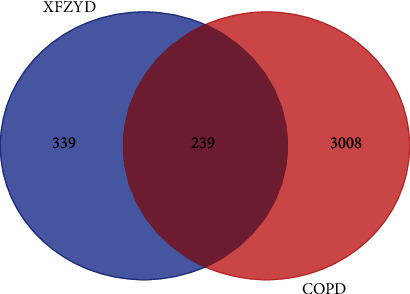
The Venn diagram of XFZYD and COPD targets.

3247 treating targets were searched and screened from ETCM and GeneCards databases after removing the duplicates. After matching the potential targets of XFZYD with the COPD-related targets, 239 overlapping targets were acquired and considered as the molecular basis of XFZYD on COPD (Figure 7).

#### 3.2.2. The Construction of PPI Network by STRING

**Figure 8:**
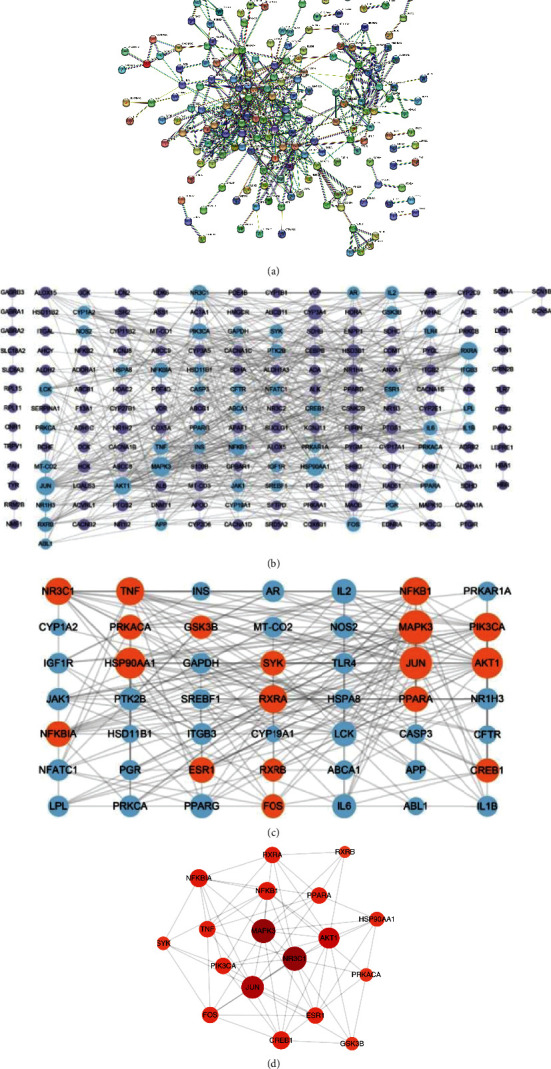
The PPI network of XFZYD on COPD. (a) The PPI network obtained from STRING database. (b) The PPT network analyzed by Cytoscape. The node genes with blue color were major genes. (c) The PPI network including the 49 major node genes. The node genes with blue color were core genes. (d) The PPI network including the 19 core node genes. The deeper the color of the node, the larger its degree value.

239 targets were uploaded to STRING database and analyzed by setting the highest confidence (Figure 8(a)). The PPI network, containing 183 nodes and 495 edges, was further downloaded and imported to Cytoscape (Figure 8(b)). By calculating three topological parameters, 49 node targets were identified as major targets of XFZYD on COPD (Figure 8(c)). Based on the results of further evaluation, 18 node targets were finally recognized as the core target genes for XFZYD in the treatment of COPD (Figure 8(d)).

#### 3.2.3. Results of GO and KEGG Pathway Enrichment Analysis

**Figure 9:**
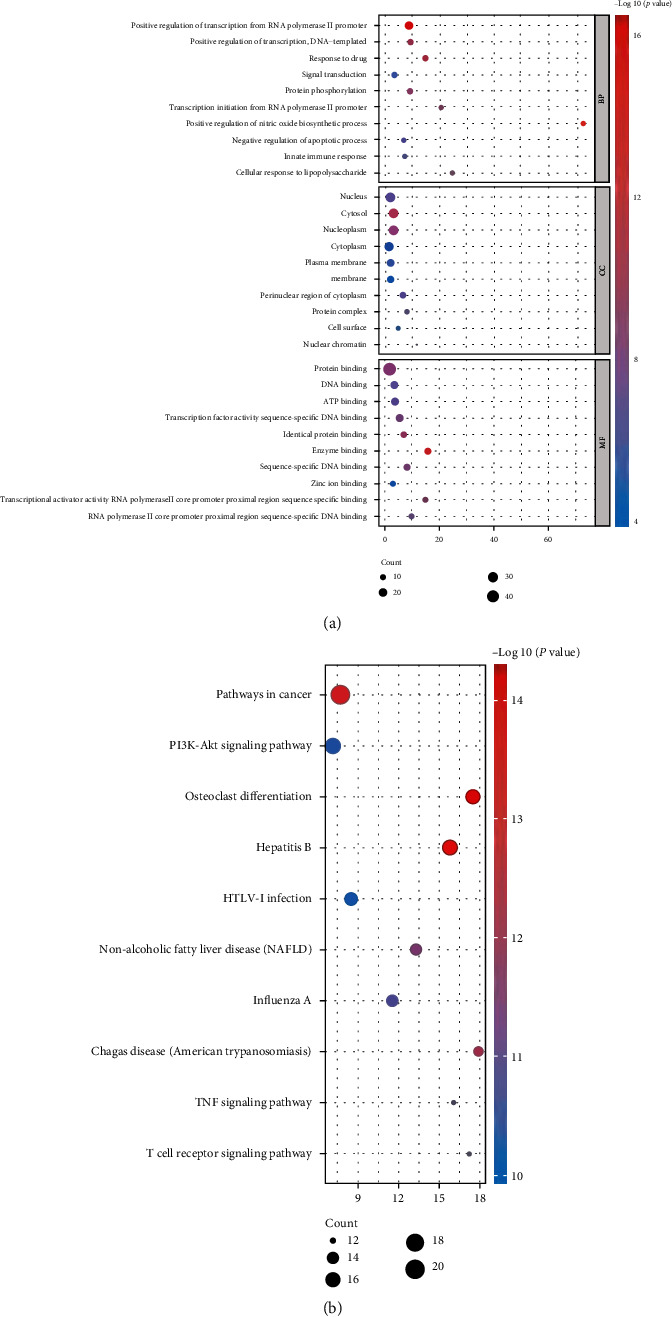
The GO and KEGG pathway enrichment analysis results of 49 major node genes. (a) The top 10 GO enrichment analysis results. (b) The top 10 KEGG pathway enrichment analysis results.

49 major targets were uploaded to the DAVID database for GO and KEGG enrichment analysis. The top 10 terms in biological process were positive regulation of transcription from RNA polymerase II promoter, positive regulation of transcription, DNA-templated, response to drug, protein phosphorylation, signal transduction, positive regulation of nitric oxide biosynthetic process, transcription initiation from RNA polymerase II promoter, innate immune response, negative regulation of apoptotic process, and cellular response to lipopolysaccharide (Figure 9(a)). In terms of cell components, the targets of XFZYD on COPD were mainly concentrated in the nucleus, cytosol, nucleoplasm, cytoplasm, plasma membrane, membrane, perinuclear region of cytoplasm, protein complex, cell surface, and nuclear chromatin (Figure 9(a)). In terms of molecular functions, the major target genes were focused on protein binding, DNA binding, ATP binding, enzyme binding, identical protein binding, transcription factor activity, sequence-specific DNA binding, transcriptional activator activity, RNA polymerase II core promoter proximal region sequence-specific binding, RNA polymerase II core promoter proximal region sequence-specific DNA binding, and zinc ion binding (Figure 9(a)).

The KEGG pathway enrichment results indicated that the major target genes of XFZYD for COPD are mainly enriched in pathways in cancer, PI3K-Akt signaling pathway, osteoclast differentiation, hepatitis B, HTLV-I infection, nonalcoholic fatty liver disease (NAFLD), influenza A, Chagas disease (American trypanosomiasis), T cell receptor signaling pathway, and TNF signaling pathway (Figure 9(b)). Based on the gene counts enriched in these pathways and the pathogenesis of COPD, the PI3K-Akt signaling pathway was regarded as the critical pathway of XFZYD in the treatment of COPD.

#### 3.2.4. Molecular Docking Results

**Figure 10:**
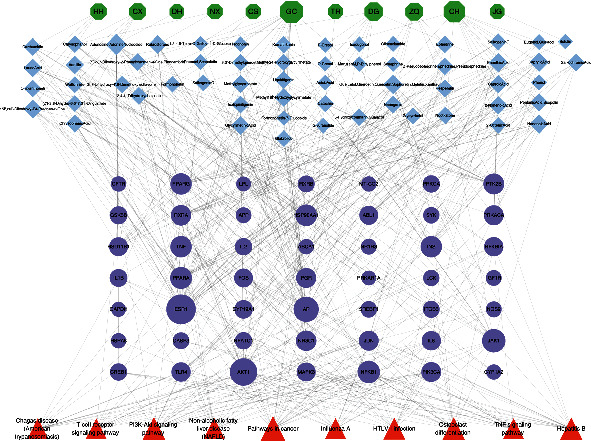
The medicine-bioactive ingredient-major target-pathway network. Green ellipse nodes represent medicines of XFZYD. Nodes with blue stand for ingredients of XFZYD. Purple ellipse nodes were target genes. Orange nodes indicate the top 10 pathways. The larger the node, the larger its degree value.

**Figure 11:**
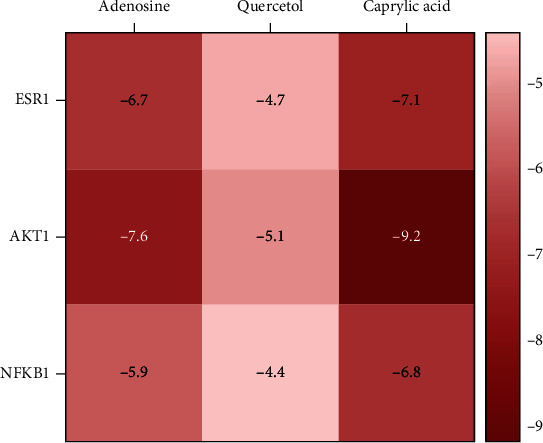
The binding energy of 3 compounds and 3 protein receptors for molecular docking.

**Figure 12:**
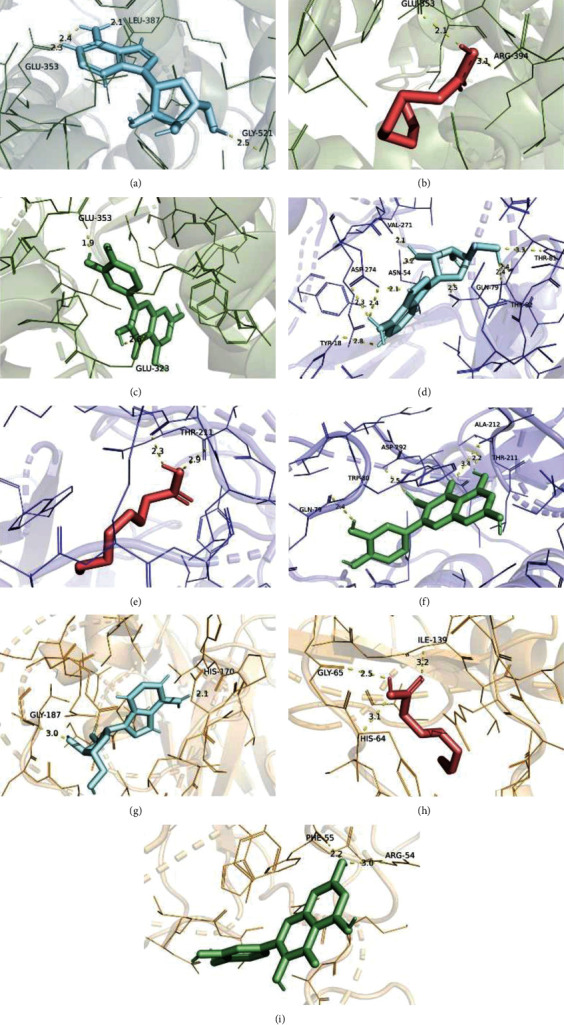
The molecular docking results visualized by PyMOL. (a) Adenosine with ESR1. (b) Quercetol with ESR1. (c) Caprylic acid with ESR1. (d) Adenosine with AKT1. (e) Quercetol with AKT1. (f) Caprylic acid with AKT1. (g) Adenosine with NFKB1. (h) Quercetol with NFKB1. (i) Caprylic acid with NFKB1.

The herb-bioactive ingredient-major target-pathway network was constructed by Cytoscape to directly represent the main molecular mechanisms of XFZYD in the treatment of COPD (Figure 10). Based on the results of herb-bioactive ingredient-major target-pathway network and PPI network, the top 3 core genes (ESR1, AKT1, and NFKB1) and top 3 ingredients, containing adenosine, quercetol, and caprylic acid, were chosen to perform the molecular docking experiments. Generally, the binding energy which is less than −4.25 kcal/mol, −5.0 kcal/mol, or−7.0 kcal/mol indicates a certain, good, or strong binding activity between ligands and receptors [50]. The docking results showed that 3 ligands and 3 receptors had a certain binding activity (Figure 11), suggesting the treating potential of XFZYD on COPD. The docking results were visualized by PyMOL (Figure 12).

## 4. Discussion

### 4.1. Clinical Evidence Summary

The 5-year survival rate for COPD patients ranges from 56% to 92% depending on the severity of the disease, which suggests that patients need early clinical interventions to control disease progression [51]. However, there is a considerable gap in the armamentarium available for treating COPD. For example, drugs that effectively control the emphysema is still scarce, but COPD patients are usually accompanied by variable degrees of emphysema [17]. Similarly, the overreliance on bronchodilators and corticosteroids leads to inevitable adverse effects, which will be detrimental to the long-term treatment of patients [52]. Notably, numerous clinical trials have provided evidence for the efficacy of XFZYD in the treatment of COPD. Therefore, the aim of the present study is to conduct a comprehensive assessment of the efficacy and safety of XFZYD on COPD.

13 original studies comprising 893 patients were included in this meta-analysis. FEV1/FVC (%), a direct indicator of pulmonary function, has been the primary measure used to diagnose COPD and determine disease severity [53]. Likewise, both FEV1 and FEV1 (%) values are important indicators for diagnosing COPD [54]. Therefore, the levels of FEV1/FVC (%), FEV1, and FEV1 (%) were used as the primary outcomes to evaluate the therapeutic effect of XFZYD on COPD. The results of our meta-analysis showed that the treatment with XFZYD significantly restored the FEV1/FVC (%), FEV1, and FEV1 (%) levels in COPD patients compared with the symptomatic supportive treatments alone, suggesting that XFZYD contributed to the recovery of pulmonary function in COPD patients. Another primary outcome, the clinical effective rate, mainly reflects whether COPD patients respond to the treatment with XFZYD. All of the included original literature which evaluated the clinical effective rate are based on the guidelines for clinical studies of new Chinese medicines. Similarly, the results demonstrated the therapeutic effect of XFZYD on COPD. Overall, the use of XFZYD on the basis of symptomatic supportive treatments significantly improved the response of COPD patients and their pulmonary function, which suggested better clinical outcomes.

Decreased *p*(O_2_) and increased *p*(CO_2_) are frequent clinical events in patients with COPD, which is strongly linked to hypoventilation, with imbalance of ventilation and perfusion being secondary factors [55]. Moreover, the presence of such hypoxemia and hypercapnia may lead to arrhythmias and pulmonary hypertension and even increase the mortality of COPD patients. Remarkably, XFZYD contributed to the recovery of *p*(O_2_) and the reduction of *p*(CO_2_) in COPD patients. As a result, the early intervention with XFZYD may help to reduce numerous adverse outcomes associated with hypoxemia and hypercapnia in COPD.

In addition to the known serious respiratory consequences, COPD is thought to significantly increase the risk of venous embolism and cardiovascular diseases, which is associated with the development of a hypercoagulable state in COPD patients and may further lead to atherosclerotic thrombosis and venous thrombosis [56, 57]. As a dissolution product of cross-linked fibrin, elevated D-dimer levels predict an increased propensity for intravascular coagulation [58]. FIB is a recognized important risk factor for cardiovascular diseases such as thrombosis and atherosclerosis. Therefore, D-dimer and FIB are considered as critical biomarkers for the assessment of coagulation and fibrinolytic activity [59]. The results of the present meta-analysis suggested that XFZYD was capable of significantly reducing D-dimer and FIB levels in COPD patients.

Generally, the cascade of coagulation consists of two pathways. The intrinsic pathway includes coagulation factors I (fibrinogen), II (prothrombin), IX, X, XI, and XII. Another pathway, the exogenous pathway, mainly involves coagulation factors I, II, VII, and X. PT responds to the activation time of the extrinsic pathway, and APTT responds to the intrinsic-pathway activation time [57]. The results of meta-analysis showed that XFZYD prolonged PT levels in COPD patients, but the effect on APTT levels was not significant, implying that XFZYD may further regulate the hypercoagulable state in COPD patients by alleviating the intrinsic pathway of coagulation to reduce the occurrence of cardiovascular diseases.

AECOPD are usually triggered by bacterial or viral infections of the respiratory tract. Significant airway inflammation, mucus secretion and gas trapping, and even systemic inflammation are present during AECOPD compared to the stable phase [60, 61]. Frequent AECOPD events can significantly reduce pulmonary function, affect quality of life, and increase the risk of death of patients [62]. With AECOPD, the medications used in the stable phase are no longer applicable for the clinical management of patients, and therefore, the 2017 Global Initiative for Chronic Obstructive Lung Disease (GOLD) document defines AECOPD as “an acute worsening of respiratory symptoms that leads to additional treatment” [63]. As a consequence, research on AECOPD treatment strategies is also exigent. The results of our subgroup analysis showed that XFZYD helped improve pulmonary function and gas retention (hypoxemia and hypercapnia) in patients, suggesting the potential of XFZYD as an adjunctive treatment for AECOPD.

The incidence of adverse events is an important indicator to evaluate the safety of treatment approaches. In included original studies, only four trials reported adverse events. For specific adverse events, only one study mentioned transient gastrointestinal symptoms in the control group, and no original study mentioned adverse events after the use of XFZYD.

Although our meta-analysis provided some clinical evidence for the treatment of COPD with XFZYD, the significant heterogeneity among studies suggested that the strength of the evidence should be viewed with caution. Basically, the clinical and methodological aspects are important factors that contribute to the heterogeneity between studies [64]. The strict inclusion and exclusion criteria have been adopted to avoid the clinical heterogeneity among the included literature in our study; however, there are still some factors which may have an influence on clinical heterogeneity that cannot be prevented in TCM-based meta-analysis. For example, the syndrome differentiation and treatment are the basic principle of TCM application in clinical practice, which means that the physicians will still add or subtract some TCMs depending on the symptoms and signs of patients possibly even if a uniform treatment protocol is prescribed in advance [65]. Moreover, the same TCMs may be used in different dosages in the treatment periods. As a consequence, these phenomena may contribute to potential differences in treatment measures between included clinical trials. In addition, the different herbal dosage forms in separate studies are also an influential factor that cannot be ignored [66]. Notably, the studies conducted by Li et al. [27] and Wu [39] applied the capsule formulation of XFZYD, whereas the remaining included studies used decoction tonics, which may also be a source of clinical heterogeneity. The methodological heterogeneity is particularly evident in the meta-analysis of TCM, which is consistent with the risk of bias assessment and GRADE evaluation in our study. A large number of TCM clinical trials are characterized by low methodological quality and low reporting quality, especially reflected in the lack of attention to randomization, allocation concealment, and blinding [67]. As a result, the methodology improvement and harmonization of clinical measures may be an important means to improve the clinical evidence in TCM and to make it more convincing.

### 4.2. Molecular Mechanisms Based on Network Pharmacology

The pharmacological activities of XFZYD have been studied to some extent, including but not limited to anti-inflammatory, tissue protection, and antiapoptotic [22, 68, 69]. Notably, numerous studies have focused on exploring the treatment potential and mechanisms of XFZYD on cardiovascular diseases, such as traumatic brain injury and coronary heart disease but with little concentration on COPD. In the current study, 293 common target genes were found between XFZYD and COPD, indicating the molecular basis of treating potential of XFZYD. According the analysis results of the herb-bioactive ingredient-major target-pathway network, *Gan Cao*, *Dang Gui*, and *Chai Hu* showed the highest degree values, which suggested that they may play necessary roles in XFZYD on COPD. *Gan Cao* has been shown to exert expectorant and pulmonary function relief effects [70]. Several components in *Gan Cao*, including isoliquiritigenin, glycyrrhizic acid, and flavonoids, have been proven for their therapeutic potentials on COPD with significant anti-inflammatory and pulmonary tissue protective effects in preclinical studies [71, 72]. *Dang Gui* is receiving attention due to its anti-inflammatory, antifibrotic, and antioxidant activities [73]. *Dang Gui* and its active ingredients have been demonstrated to have therapeutic effects for some respiratory diseases, such as pulmonary fibrosis, asthma, and lung injury in preclinical researches, suggesting that it may have therapeutic potential on COPD [74–76]. Another medicinal, *Chai Hu*, has also been shown to have anti-inflammatory effects and its polysaccharides can improve the lung injury in vivo [77, 78].

Adenosine, quercetol, and caprylic acid were identified as the key ingredients of XFZYD and were used for molecular docking experiments. Adenosine is a nucleoside signaling molecule produced by cells when differing stress or damage [79]. After being produced, adenosine interacts with its receptors to activate adenosine-related signal, further regulating the cell functions [80]. The adenosine signal is believed to participate in the occurrence and development of COPD [81]. Moreover, experts suggest that the adenosine receptors may be the treating targets of COPD [82]. However, signals induced by different adenosine receptors will exert proinflammatory or anti-inflammatory properties in pulmonary inflammatory diseases, which means that whether adenosine exogenously intake via XFZYD has a therapeutic effect on COPD still needs to be further evaluated [80]. Quercetol, commonly named quercetin, its therapeutic potential on COPD has been proven by preclinical and clinical studies [83, 84]. The pharmacological mechanisms are related to its anti-inflammatory and antioxidant effects [85]. Furthermore, an in vitro study based on mononuclear cells from COPD patients showed that quercetin can increase the cellular sensitivity to steroids, which is clinically important for steroid-resistant COPD patients [86]. Caprylic acid is a medium chain fatty acid derived mainly from coconut oil and goat milk and is also applied as an antimicrobial agent in industry production [87]. Current researches have revealed its advantages in terms of energy homeostasis, neuroprotection, hypoglycemia, and even antitumor, suggesting its clinical application capability [88]. However, its therapeutic advantages on COPD and specific mechanisms apparently remain uncovered.

The pathogenesis of COPD is a complex progress containing various inflammatory cells, inflammatory mediators, and cell signaling pathways [89]. Excessive oxidative stress and inflammatory responses are necessary factors in COPD pathogenesis [90, 91]. It is worth noting that the dysregulation of the PI3K/AKT pathway plays an important role in the activation of inflammatory cells and release of inflammatory mediators in COPD [92]. In addition, the activation of Nrf2, mainly induced by the PI3K/AKT pathway, also regulates the oxidative stress and chronic inflammation in COPD [93]. In preclinical investigations, the intervention of the PI3K/AKT pathway has shown the advantages in improving chronic inflammation in COPD, suggesting that the PI3K/AKT pathway may be the hopeful treating target [94, 95]. In our study, the influence on the PI3K/AKT signaling pathway was recognized as key mechanism of XFZYD based on the KEGG pathway enrichment analysis, which provides a vision to fully reveal the mechanisms of XFZYD in the treatment of COPD. In our future research, chemical analysis and animal studies can be combined with network pharmacology to comprehensively identify the key active ingredients and validate the complex mechanisms of XFZYD in the treatment of COPD, which will provide stronger evidence of its clinical application.

### 4.3. The Communication between Clinical Evidence and Network Pharmacology Results

The application of XFZYD is contributed to the improved pulmonary function, relieved ventilatory disturbances, and corrected hypercoagulability of COPD patients in an evidence-based medicine view. Moreover, the network pharmacology results suggested that XFZYD is capable of intervening with the PI3K/AKT pathway to improve COPD. Thus, in reality, the oxidative stress and inflammatory damage are critical aspects of the numerous pathogenic mechanisms of COPD. The risk factors, such as cigarette smoke, lead to the production of numerous reactive oxygen species (ROS), which further leads to lung cell damage, alveolar epithelial damage, and excessive mucus secretion [96]. At the same time, ROS can promote the expression of inflammatory mediators and aggravate lung tissue damage, leading to impaired lung function in patients [89]. Furthermore, the chronic immune cell infiltration and inflammatory mediator release promote the secretion of proteases and growth factors, which promote the deleterious changes in epithelial, stromal, and parenchymal cells and induce airway remodeling, leading to airway obstruction and impaired ventilation in COPD patients [97]. In addition, ROS and inflammatory mediators can also increase the production of coagulation factors, activate the coagulation system, and lead to the hypercoagulable state of patients [57].

It has been shown that activation of the PI3K/AKT pathway is associated with oxidative stress, release of inflammatory mediators, and airway remodeling in COPD [92]. Several PI3K protein inhibitors have been shown to reduce the ROS production and inflammatory cell infiltration and to modulate protease dysregulation to mitigate COPD progression in clinical or preclinical studies [98–100]. XFZYD effectively treats COPD by intervening in the PI3K/AKT pathway, ameliorating oxidative stress and inflammatory responses, relieving the airway remodeling and ventilation disorders, correcting hypercapnia and hypoxemia, and alleviating hypercoagulability of patients, suggesting the consistency between the results of meta-analysis and network pharmacology predictions and revealing an important mechanism for the treatment of COPD with XFZYD.

### 4.4. Strengths and Limitations

As far as we know, this is the first systematic and comprehensive evaluation and exploration of the efficacy and molecular mechanisms of XFZYD in the treatment of COPD. Some alternative medicine approaches usually are not taken into consideration in clinical practice because of the lack of evidence of efficacy and unclear mechanisms of action. Notably, the meta-analysis and network pharmacology techniques have become well-established tools for investigating the disease treatment strategies. Such researches can help to reveal the therapeutic potential and advantages of complementary and alternative medicine, especially TCM, for various diseases. In our study, the combination of meta-analysis and network pharmacology may provide more specific information about the results of clinical trials, that is, the molecular mechanism of XFZYD for COPD, which will more rationally explain why XFZYD is a potential strategy for COPD. Moreover, it will also facilitate the subsequent research of XFZYD and even the development work of potent drugs for COPD. As a consequence, a research model that uses meta-analysis and network pharmacology in combination is a worthy advocating tool to explore the clinical potential and mechanisms of action of treatment options, especially in alternative and complementary medicine fields.

Our current study still has limitations. Firstly, the lack of high-quality, multicenter, randomized controlled studies and the fact that some of the included original studies did not mention the details of random assignment principles and blinding may reduce the quality of the present meta-analysis. Secondly, the absence of original studies using XFZYD alone as the treatment group and placebo as the control group resulted in the inability to assess the clinical efficacy of XFZYD alone in the treatment of COPD. Finally, due to the inadequate reporting of adverse events, we were unable to conduct a comprehensive evaluation of the safety of XFZYD on COPD. More high-quality, multicenter, rigorous RCTs are demanded to investigate the efficacy and safety of XFZYD in the treatment of COPD in further research. Finally, the exploration of molecular mechanisms based on network pharmacology is contributed to reveal the multicomponent, multitarget, and multipathway basis of XFZYD in the treatment of COPD. However, the experiments with animal models are still needed to provide stronger evidence for analysis results of network pharmacology, which is our future direction.

## 5. Conclusion

XFZYD may be a potential adjuvant therapeutic strategy for the treatment of COPD. Intervention with XFZYD is beneficial in restoring pulmonary function and improving gas retention and coagulation disorders in COPD patients in clinical practice. The early use of XFZYD during the stable phase and AECOPD may help patients to reduce the appearance of dangerous clinical outcomes. XFZYD, which has many active ingredients, determines its multitarget and multipathway mechanism in the treatment of COPD, and the intervention of the PI3K/AKT pathway may be the critical mechanism. In conclusion, this study provides some evidence for the clinical application of XFZYD.

## Figures and Tables

**Table 1:** The characteristics of the original studies included in the meta-analysis.

Authors	No. of patients		Age of patients		Disease course		Stage	Outcomes	Adverse events		References
	Treatment	Control	Treatment	Control	Treatment	Control			Treatment	Control	
Li et al. 2021 (a)	30	30	74.80 ± 9.49	76.50 ± 7.10	15.63 ± 7.51	17.30 ± 4.52	Stable COPD	(1)(2)(3)(5)(6)(7)(8)(9)(10)	0	0	[27]
Li et al. 2021 (b)	30	30	75.77 ± 9.27	76.37 ± 5.81	14.90 ± 6.64	17.47 ± 3.28	AECOPD	(1)(2)(3)(5)(6)(7)(8)(9)(10)	0	2	[27]
Li 2019	25	25	63.7 ± 4.5	63.7 ± 4.3	9.0 ± 2.3	8.5 ± 2.0	Stable COPD	(1)(3)(4)	NR	NR	[42]
Liu et al. 2018	33	30	63 ± 6.2	64 ± 5.5	16 ± 2.4	15 ± 2.7	AECOPD	(2)(3)(4)(5)(6)(7)(9)(10)	NR	NR	[45]
Li et al. 2017	40	40	61.63 ± 5.14	62.39 ± 4.32	13.73 ± 6.83	13.23 ± 7.54	Stable COPD	(1)(3)(4)	0	0	[47]
Ma 2016	32	32	63.18 ± 5.02	62.5 ± 4.49	10.3 ± 2.45	9.42 ± 3.72	AECOPD	(1)(3)(4)	NR	NR	[41]
Mao 2016	29	29	67.17	17.47	NR	NR	Stable COPD	(2)(3)	NR	NR	[38]
Fang et al. 2015	44	46	65.0 ± 4.8	66.0 ± 5.9	10.2 ± 3.3	12.5 ± 4.0	AECOPD	(1)(3)(5)(6)(7)(8)(10)	0	0	[43]
Li 2015	30	30	59.5 ± 4.42	58.6 ± 4.63	9.55 ± 3.53	9.17 ± 3.48	AECOPD	(1)(2)(3)(4)(5)(6)	NR	NR	[49]
Cai et al. 2014	24	22	50~76	50~75	7-26	7-26	NR	(4)	NR	NR	[44]
Zhang 2014	30	30	61.9 ± 5.7	62.1 ± 5.3	14.2 ± 3.9	13.4 ± 4.6	AECOPD	(1)(3)(8)	NR	NR	[40]
Hong et al. 2013	30	30	72.1 ± 9.3	74.5 ± 8.8	NR	NR	AECOPD	(4)(7)	NR	NR	[48]
Wang et al. 2011	31	31	NR	NR	NR	NR	AECOPD	(4)	NR	NR	[46]
Wu 2011	40	40	67.2	66.8	NR	NR	Stable COPD	(1)(2)(5)(6)	NR	NR	[39]

(1): FEV1; (2): FEV1%; (3): FEV1/FVC (%); (4): clinical effective rate; (5): *p*(O_2_); (6): *p*(CO_2_); (7): D-dimer; (8): FIB; (9): PT; (10): APTT; AECOPD: acute exacerbation of chronic obstructive pulmonary disease; NR: not reported; COPD: chronic obstructive pulmonary disease.

## Data Availability

The original contributions presented in the study are included in the article/supplementary materials; further inquiries can be directed to the corresponding author.

## Supplementary Materials

Supplementary 1 Supplementary Table 1: the searching details in each database. Supplementary Table 2: the specific dosages of XFZYD and the treatment strategies of the control group in each study.

Supplementary 2 Supplementary Figure 1: the sensitivity analysis results of the FEV1/FVC (%).

Supplementary 3 Supplementary Figure 2: the quality of evidence assessment by GRADE profiler.
