# Supplementary material for: Analysis of the Clinical Efficacy and Molecular Mechanism of Xuefu Zhuyu Decoction in the Treatment of COPD Based on Meta-Analysis and Network Pharmacology
Source: Comput Math Methods Med. 2022 Nov 26;2022:2615580. doi: 10.1155/2022/2615580 (PMC9720234; doi:10.1155/2022/2615580)
Supplement: Supplementary 1 — Supplementary Table 1: the searching details in each database. Supplementary Table 2: the specific dosages of XFZYD and the treatment strategies of the control group in each study. [file 2615580.f1.docx]

Supplementary Material

# Supplementary Tables

Supplementary table 1: The searching details in each database

| Database | Searching details |
| --- | --- |
| CNKI | TKA='xuefu zhuyu' and TKA=' chronic obstructive pulmoriary disease '+'COPD' |
| Wanfang | Title/Keywords:(xuefu zhuyu) and Title/Keywords:( chronic obstructive pulmoriary disease or COPD) |
| CQVIP | M=xuefu zhuyu AND M=(chronic obstructive pulmoriary disease OR COPD） |
| CBM | Xuefu zhuyu AND (COPD OR chronic obstructive pulmoriary disease) |
| Pubmed  Embase | ((xuefu zhuyu decoction[Title/Abstract]) OR (xuefuzhuyu decoction[Title/Abstract]) OR (xuefu zhuyu[Title/Abstract]) OR (xuefuzhuyu[Title/Abstract])) AND ((COPD[Title/Abstract]) OR (chronic obstructive pulmoriary disease[Title/Abstract])) |
| WOS | TS=(Xuefu zhuyu decoction OR xuefuzhuyu decoction OR xuefu zhuyu OR xuefuzhuyu) AND TS=(chronic obstructive pulmoriary disease OR COPD) |
| Cochrane library | Title/keyword/abstract: xuefu zhuyu decoction OR xuefuzhuyu decoction OR xuefuzhuyu OR xuefu zhuyu |

Supplementary table 2: The specific dosages of XFZYD and the treatment strategies of control group in each study.

| Study | Treatment strategies of experimental group | Source of medicinals in experimental group | Treatment strategies of control group |
| --- | --- | --- | --- |
| Li et al. 2021(a) | Xuefu Zhuyu Capsules + treatment strategies of control group | Tianjin Hongrentang Pharmaceutical Co., Ltd | Tiotropium Bromide, 18μg a time, once a day  Budesonide/Formotero, 4.5μg a time, twice a day |
| Li et al. 2021 (b) | Xuefu Zhuyu Capsules + treatment strategies of control group | Tianjin Hongrentang Pharmaceutical Co., Ltd | Low-flow oxygen inhalation, phlegm relieving cough and asthma, anti-infective drugs, bronchodilators and glucocorticoid were given |
| Li 2019 | The dried ripe seed of *Prunus persica (L.)* Batsch *(Tao Ren),* 5g; the dried flower of *Carthamus tinctorius* L. (*Hong Hua*) 9g, the dried root of *Angelica sinensis* (Oliv.) Diels (*Dang Gui*), 9g; the fresh or dried tuberous root of *Rehmannia glutinosa* (Gaertn.) DC. (*Sheng Di*), 9g; the dried rhizome of *Conioselinum anthriscoides 'Chuanxiong'* (*Chuan Xiong*), 5g; the dried root of *Paeonia lactiflora* Pall. (*Chi Shao*), 6g; the dried unripe fruit of *Citrus × aurantium* L. (*Zhi Qiao*), 5g; the dried root of *Bupleurum chinense* DC. (*Chai Hu*), 6g; the dried root of *Achyranthes bidentata* Blume (*Niu Xi*),10g; the dried root of Platycodon grandiflorus (Jacq.) A.DC. (*Jie Geng*), 10g; the dried root and rhizome of *Glycyrrhiza glabra* L. (*Gan Cao*), 6g; the dried root of *Astragalus mongholicus* Bunge (*Huang Qi),* 20g; and the dried root bark of *Morus alba* L. (*Sang Bai Pi),* 20g+ treatment strategies of control group | Prepared by Li | Tiotropium powder inhalation, 18 μg a time, once a day.  Doxofylline tablets, 0.2g a time, twice a day. |
| Liu et al. 2018 | *Tao Ren,* 15g; *Hong Hua,* 9g; *Dang Gui,* 9g; *Sheng Di,* 6g; *Chuan Xiong,* 6g; *Chi Shao,* 10g; *Zhi Qiao,* 8g; *Chai Hu,* 10g; *Niu Xi,* 8g; *Jie Geng,* 6g; *Gan Cao,* 8g; the dried root of *Codonopsis pilosula* (Franch.) Nannf.(*Dang Shen),* 15g; and *Huang Qi* 20g+ treatment strategies of control group | Prepared by Liu et al. | Anti - infection, oxygen inhalation, cough and asthma |
| Li et al. 2017 | *Tao Ren,* 5g; *Hong Hua,* 9g; *Dang Gui,* 9g; *Sheng Di,* 9g; *Chuan Xiong,* 4g; *Chi Shao,* 6g;, *Zhi Qiao,* 6g; *Chai Hu,* 6g; *Niu Xi,* 9g; *Jie Geng,* 10g; *Gan Cao,* 6g; *Huang Qi,* 20g; and *Sang Bai Pi* 20g+ treatment strategies of control group | Prepared by Li et al. | Tiotropium powder inhalation, 18 μg a time, once a day.  Doxofylline tablets, 0.2g a time, twice a day. |
| Ma 2016 | *Tao Ren,* 12g; *Hong Hua,* 6g; *Dang Gui,* 9g; *Sheng Di,* 9g; *Chuan Xiong,* 4.5g; *Chi Shao,* 6g; *Zhi Qiao,* 6g; *Chai Hu,* 3g; *Niu Xi,* 9g; *Jie Geng,* 4.5g; and *Gan Cao* 6g+ treatment strategies of control group | Prepared by Ma. | Oxygen absorption, anti - infection, anti - inflammation, bronchiectasis |
| Mao 2016 | *Tao Ren,* 5g; *Hong Hua,* 10g; *Dang Gui,* 6g; *Sheng Di,* 5g; *Chuan Xiong,* 6g; *Chi Shao,* 10g; *Zhi Qiao,* 6g; *Chai Hu,* 5g; *Niu Xi,* 12g; *Jie Geng,* 5g; and *Gan Cao* 6g + treatment strategies of control group | Prepared by Mao. | Carbocisteine Tablets, 0.35g/table, two tablets a time, three times a day.  Tiotropium Bromide Powder for Inhalation, 18 μg a time, once a day. |
| Fang et al. 2015 | *Tao Ren,* 12g; *Hong Hua,* 15g; *Dang Gui,* 10g; *Sheng Di,* 12g; *Chuan Xiong,* 10g; *Chi Shao,* 9g; *Zhi Qiao,* 6g; *Chai Hu,* 6g; *Niu Xi,* 12g; *Jie Geng,* 9g; *Gan Cao,* 3g; and the dried mature fruit og *Trichosanthes kirilowii* Maxim.(*Gua Lou)* 5g + treatment strategies of control group | Prepared by Fang et al. | Anti-infection, continuous low-flow oxygen inhalation to improve, asthma, correction of water and electrolyte disorders and nutritional support |
| Li 2015 | *Tao Ren,* 15g; *Hong Hua*, 10g; *Dang Gui,* 10g; *Sheng Di,* 9g; *Chuan Xiong,* 5g; *Chi Shao,* 6g; *Zhi Qiao,* 6g; *Chai Hu,* 3g; *Niu Xi,* 10g; *Jie Geng,* 5g; and *Gan Cao* 6g+ treatment strategies of control group | Prepared by Li. | Low flow oxygen inhalation  Ceftazidime 2.0 g intravenously, twice a day  Polytheophylline 0.3g intravenous infusion, once a day  Ambroxol 30mg intravenously, twice daily |
| Cai et al. 2014 | *Tao Ren,* 12g; *Hong Hua,* 9g; *Dang Gui,* 9g; *Sheng Di,* 9g; *Chuan Xiong,* 3g; *Chi Shao,* 6g; *Zhi Qiao,* 6g; *Chai Hu,* 3g; *Niu Xi,* 9g; *Jie Geng,* 9g; and *Gan Cao* 3g+ treatment strategies of control group | Prepared by Cai et al. | Intermittent oxygen inhalation, anti - infection, phlegm, spasmolysis and asthma |
| Zhang 2014 | *Tao Ren,* 15g; *Hong Hua,* 12g; *Dang Gui,* 15g; *Sheng Di,* 15g; *Chuan Xiong,* 10g; *Chi Shao,* 15g; *Zhi Qiao,* 12g; *Chai Hu,* 6g; *Niu Xi,* 12g; *Jie Geng,* 5g; *Gan Cao,* 6g; and *Huang Qi* 30g + treatment strategies of control group | Prepared by Zhang. | Anti-infection, cough, phlegm, spasmolysis and asthma, anti-inflammation, strengthening camp and low flow oxygen inhalation |
| Hong et al. 2013 | *Tao Ren,* 12g; *Hong Hua,* 6g; *Dang Gui,* 9g; *Sheng Di,* 9g; *Chuan Xiong,* 3g; *Chi Shao,* 6g; *Zhi Qiao,* 9g; *Chai Hu,* 3g; *Niu Xi,* 9g; *Jie Geng,* 9g; and *Gan Cao* 3g+ treatment strategies of control group | Prepared by Hong et al. | Controlled low flow oxygen therapy, anti-infection, phlegm, spasmolysis, antiasthmatic |
| Wang et al. 2011 | *Tao Ren,* 12g; *Hong Hua,* 6g; *Dang Gui,* 9g; *Sheng Di,* 9g; *Chuan Xiong,* 9g;*Chi Shao,* 6g; *Zhi Qiao,* 9g; *Chai Hu,* 6g; *Niu Xi,* 12g; *Jie Geng,* 6g; and *Gan Cao* 3g+ treatment strategies of control group | Prepared by Wang et al. | Oxygen inhalation, anti-infection, bronchial dilation, and using glucocorticoid |
| Wu 2011 | Xuefu Zhuyu Capsules + treatment strategies of control group | Tianjin Hongrentang Pharmaceutical Co., Ltd | Aminophylline Sustained-release Tablets, 0.1g a time, twice a day.  Inhaling salbutamol aerosol, twice a day. Home oxygen therapy, respiratory muscle exercise |
