# Supplementary figures and images for: Analysis of the Clinical Efficacy and Molecular Mechanism of Xuefu Zhuyu Decoction in the Treatment of COPD Based on Meta-Analysis and Network Pharmacology
Source: Comput Math Methods Med. 2022 Nov 26;2022:2615580. doi: 10.1155/2022/2615580 (PMC9720234; doi:10.1155/2022/2615580)

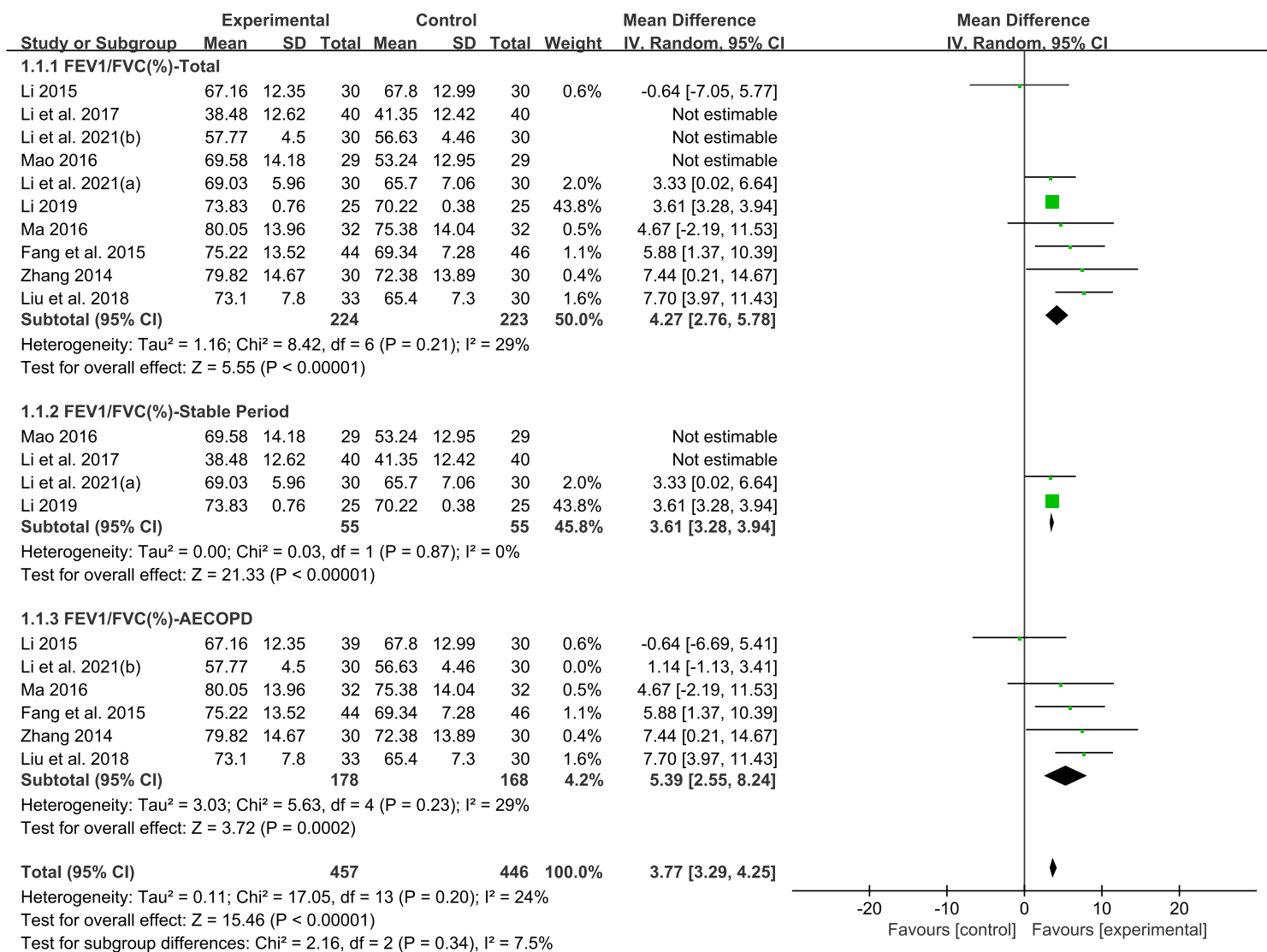

Supplement: Supplementary 2 — Supplementary Figure 1: the sensitivity analysis results of the FEV1/FVC (%). [file 2615580.f2.pdf]
