# Supplementary material for: Analysis of the Clinical Efficacy and Molecular Mechanism of Xuefu Zhuyu Decoction in the Treatment of COPD Based on Meta-Analysis and Network Pharmacology
Source: Comput Math Methods Med. 2022 Nov 26;2022:2615580. doi: 10.1155/2022/2615580 (PMC9720234; doi:10.1155/2022/2615580)
Supplement: Supplementary 3 — Supplementary Figure 2: the quality of evidence assessment by GRADE profiler. [file 2615580.f3.pdf]

Xuefu Zhuyu Decoction combined with symptomatic supportive treatments for COPD

Patient or population: patients with COPD
Settings:
Intervention: Xuefu Zhuyu Decoction combined with symptomatic supportive treatments

Outcomes	Illustrative comparative risks* (95% CI)		Relative effect (95% CI)	No of Participants (studies)	Quality of the evidence (GRADE)	Comments
	Assumed risk	Corresponding risk				
	**Control**	**Xuefu Zhuyu Decoction combined with symptomatic supportive treatments**				
**FEV1/FVC(%) - FEV1/FVC(%)-Total**		The mean fev1/fvc(%) - fev1/fvc(%)-total in the intervention groups was **4.12 higher** (2.09 to 6.15 higher)		645 (10 studies)	⊕⊕⊝⊝ **low**[1,2]	
**FEV1% - FEV1%-Total**		The mean fev1% - fev1%-total in the intervention groups was **7.33 higher** (2.38 to 12.27 higher)		381 (6 studies)	⊕⊕⊝⊝ **low**[1,2]	
**FEV1 - FEV1-Total**		The mean fev1 - fev1-total in the intervention groups was **0.16 higher** (0.06 to 0.26 higher)		604 (9 studies)	⊕⊕⊝⊝ **low**[1,2,3]	
**P(O2) - P(O2)-Total**		The mean p(o2) - p(o2)-total in the intervention groups was **8.38 higher** (4.88 to 11.88 higher)		413 (6 studies)	⊕⊕⊝⊝ **low**[1,2]	
**P(CO2) - P(CO2)-Total**		The mean p(co2) - p(co2)-total in the intervention groups was **3.43 lower** (5.57 to 1.28 lower)		413 (6 studies)	⊕⊕⊝⊝ **low**[1,2]	
**Clinical efficacy rate - Clinical efficacy rate**	**Study population**		**RR 1.26** (1.17 to 1.37)	485 (8 studies)	⊕⊕⊕⊝ **moderate**[1]	
	**746 per 1000**	**940 per 1000** (873 to 1000)				
	**Moderate**					
	**724 per 1000**	**912 per 1000** (847 to 992)				
**Adverse events**	**Study population**		**OR 0.19** (0.01 to 4.06)	290 (4 studies)	⊕⊝⊝⊝ **very low**[1,3]	
	**14 per 1000**	**3 per 1000** (0 to 53)				
	**Moderate**					
	**0 per 1000**	**0 per 1000** (0 to 0)				

**D-dimer**		The mean d-dimer in the intervention groups was **2.41 standard deviations lower** (3.98 to 0.84 lower)		333 (5 studies)	⊕⊕⊝⊝ **low**[1,2]	SMD -2.41 (-3.98 to -0.84)
**FIB**		The mean fib in the intervention groups was **1.45 lower** (2.16 to 0.74 lower)		270 (4 studies)	⊕⊕⊝⊝ **low**[1,2]	
**PT**		The mean pt in the intervention groups was **3.01 higher** (2.51 to 3.52 higher)		183 (3 studies)	⊕⊕⊕⊝ **moderate**[1,2]	
**APTT**		The mean aptt in the intervention groups was **1.46 higher** (1.15 lower to 4.07 higher)		273 (4 studies)	⊕⊝⊝⊝ **very low**[1,2,3]	

*The basis for the **assumed risk** (e.g. the median control group risk across studies) is provided in footnotes. The **corresponding risk** (and its 95% confidence interval) is based on the assumed risk in the comparison group and the **relative effect** of the intervention (and its 95% CI).

CI: Confidence interval; **RR:** Risk ratio; **OR:** Odds ratio;

GRADE Working Group grades of evidence
High quality: Further research is very unlikely to change our confidence in the estimate of effect.
Moderate quality: Further research is likely to have an important impact on our confidence in the estimate of effect and may change the estimate.
Low quality: Further research is very likely to have an important impact on our confidence in the estimate of effect and is likely to change the estimate.
Very low quality: We are very uncertain about the estimate.

[1] There are unclear risks on random sequence generation, allocation concealment, blinding of participants and personnel, blinding of outcome assessment
[2] The outcome is heterogeneous
[3] Real effects exceed the upper and lower bounds of the confidence interval
